# *In vitro* evaluation of neuroprotective effect of *Anethum graveolens* and its bioactives in H_2_O_2_‐induced oxidative stress model of SH‐SY5Y and biochemical studies

**DOI:** 10.1002/alz.090623

**Published:** 2025-01-09

**Authors:** Himadri Sharma

**Affiliations:** ^1^ Gachon University, Seongnam, Gyeonggido Korea, Republic of (South)

## Abstract

**Background:**

*Anethum graveolans* commonly known as Dill is an herb from celery family displaying anti‐oxidant benefits. The present study focused on the potential of *Anethum graveolans* as a multifunctional curative remedy for AD treatment.

**Method:**

Hexane (H) and ethyl acetate (EA) extracts of Dill were prepared and subjected to GC‐MS for identification of important bioactive components. H_2_O_2_ was used to induce oxidative stress in SH‐SY5Y cells and evaluation was done based on neuroprotection, reduced reactive oxygen species, and restoration of mitochondrial membrane potential (MMP). Additionally, anti‐Aβ fibrilization/ oligomerization activities were also analyzed along with anti‐acetylcholinesterase activity.

**Result:**

Based on GC‐MS results, 3 major components were identified apiole, carvone and dihydrocarvone (DHC). The extracts and pure compounds were assessed for their neuroprotective potential in which dose‐dependent increase in neuroprotection was observed in both extracts, apiole and carvone. Dill‐H displayed a dose‐dependent decrease in ROS levels which was more prominent than Dill‐EA extract. Pure compounds also exerted a decrease in ROS level with increasing concentration. Dill extracts (H/EA) exhibited dose‐dependent increase in mitochondrial membrane potential along with Carvone and DHC. Apiole showed less restoration of MMP compared to other pure compounds. For anti‐Aβ fibrilization activity, Dill‐EA, Carvone and Apiole show ≈40% inhibition followed by DHC (35%) and Dill‐H (30%). In MDS, maximum oligomerization inhibition was observed in DHC (26%) and apiole (14%). Dill extracts and compounds exhibit anti‐AChE activity and inhibited the enzyme through competitive inhibition except for DHC.

**Conclusion:**

We focused our research on small molecules from food to prevent AD. There is a need for a drug that is easily accessible, cost‐effective and has no/fewer side effects. In our study, Dill extracts and its identified bioactive compounds exhibited a multitargeted neuroprotection in the SH‐SY5Y cells making it a promising candidate for AD drug development.

